# DeepInsight软件术前肺部支气管血管成像的真实性研究

**DOI:** 10.3779/j.issn.1009-3419.2021.104.03

**Published:** 2021-02-20

**Authors:** 望 张, 亮 陈, 俊 王, 伟 闻, 心峰 徐, 全 朱

**Affiliations:** 210029 南京，南京医科大学第一附属医院/江苏省人民医院胸外科 Department of Thoracic Surgery, Jiangsu Provincial People' s Hospital, The First Affiliated Hospital of Nanjing Medical University, Nanjing 210029, China

**Keywords:** DeepInsight软件, 肺部计算机断层血管造影检查, 三维支气管血管成像, 肺段切除术, DeepInsight software, Lung computed tomographic angiography examination, Three-dimensional bronchial angiography, Segmentectomy

## Abstract

**背景与目的:**

精准肺段切除术已成为肺结节及早期肺癌外科治疗的首要选择，而其手术重点及难点则在于对病灶的精准定位与切除。DeepInsight为我中心与东软公司共同研发的精准肺部手术辅助软件，可术前明确肺部精确解剖，定位肺部病灶位置，此次研究即为验证DeepInsight肺部支气管血管成像辅助手术的真实性及可靠性。

**方法:**

本研究共纳入了2016年8月1日-2019年12月31日江苏省人民医院胸外科收治的1, 020例预行手术治疗患者，所有入组患者肺部结节直径 < 2.0 cm，术前均行肺结节计算机断层血管造影（computed tomographic angiography, CTA）检查，使用DeepInsight软件术前进行术侧肺部支气管血管成像，识别受累肺段、肺动脉及肺静脉，2位胸外科医师采用5分法独立评估受累肺血管的可视性情况，*χ*^2^检验评估观察者间的一致性。此外，术侧肺部血管虚拟成像及真实解剖对比，由2名胸外科主任医师最终决定受累肺血管情况。

**结果:**

1, 020例患者术前运用DeepInsight软件术侧肺部虚拟成像血管与术中受累血管的数量及空间解剖关系之间的差异无明显统计学意义，观察者间的一致性均相当满意。

**结论:**

Dee术中进行pInsight软件肺部支气管血管虚拟成像可精准具化肺部真实血管情况，辅助肺段切除术的完成。

## Abstract (en)

**Background and objective:**

Precise segmentectomy has become the first choice of surgical treatment for pulmonary nodules and early lung cancer, and the key and difficult point of the surgery lies in the precise location and resection of the lesion. DeepInsight is an auxiliary software for precise lung surgery jointly developed by our center and Neusoft Company, which can determine the precise anatomy of the lung and locate the location of lung lesions before operation. This study is to verify the authenticity and reliability of DeepInsight lung bronchial angiography assisted surgery.

**Methods:**

In this study, 1, 020 patients with pulmonary nodules < 2.0 cm in diameter were included in the Department of Thoracic Surgery Jiangsu Provincial People' s Hospital from August 1, 2016 to December 31, 2019. Computed tomographic angiography (CTA) was performed on all the included patients before surgery. The DeepInsight software was used to perform preoperative bronchial angiography on the operative side of the lung to identify the affected pulmonary segments, pulmonary arteries and pulmonary veins. Two thoracic surgeons independently assessed the visibility of the affected pulmonary vessels using the 5-point method, and the *χ*^2^ test assessed the consistency between observers. In addition, virtual imaging and real anatomy of pulmonary vessels on the operative side were performed during the operation, and the involved pulmonary vessels were finally determined by 2 chief physicians of thoracic surgery.

**Results:**

There were no statistically significant differences between the number and spatial anatomy of the vessels involved in the pulmonary virtual imaging using DeepInsight software before operation and the number of vessels involved during operation in 1, 020 patients. And the consistency among observers is quite satisfactory.

**Conclusion:**

The DeepInsight software virtual imaging of pulmonary bronchial vessels can accurately reconstruct the actual pulmonary vessels and assist the completion of pulmonary segmental resection.

随着低剂量螺旋计算机断层扫描（computed tomography, CT）筛查的推广，肺结节的就诊率呈现上升趋势，早期非小细胞肺癌（non-small cell lung cancer, NSCLC）的手术患者不断增加^[1]^，精准肺段切除术已成为肺结节及早期肺癌外科治疗的首要选择^[2]^。研究发现，对于直径 < 2 cm的早期肺癌，肺段切除具有和肺叶切除相似的远期疗效^[3, 4]^，并且最大程度地保留了患者的肺功能，减少了术后并发症^[5]^。

在肺段切除术中，最大的难题在于肺解剖结构的复杂多变以及血管的纵横交错，而使用DeepInsight软件术前进行肺部受累支气管血管虚拟成像可以重建肺血管、支气管和肺结节，辨认解剖变异和结节的解剖归属^[6-8]^，针对肺段解剖复杂和肺结节位置多样的难题设计个体化的手术方案，在肺段切除术中具有至关重要的作用^[9]^。

本研究通过江苏省胸外科平台验证DeepInsight肺部支气管血管成像辅助手术的真实性及可靠性。

## 1 资料与方法

### 1.1 临床资料

回顾性分析江苏省人民医院胸外科中心2016年8月1日-2019年12月31日1, 020例肺部结节直径 < 2.0 cm，因“肺结节”入院拟行肺段切除术的患者临床资料。术前筛除标准如下：①患者暂不适宜手术治疗，如急性感染、不稳定性心绞痛、急性脑梗塞、深静脉血栓形成、多发性肿瘤病变等；②肺部情况复杂，如既往肺部手术病史、严重慢性阻塞性肺疾病、反复发作的气胸、既往结核病史、既往脓胸/胸膜炎病史、解剖变异如肺隔离症/支气管畸形及肺纤维化等；③患者或家属拒绝手术治疗，或其他原因未手术的。最后，对1, 020例患者的肺部支气管血管成像与术中录像进行对比研究。

### 1.2 肺部CTA检查

检查方法采用Siemens Sensation 64层螺旋CT机，MEDRAD STELLANT高压注射器。扫描参数: 管电压120 KV，采用自动管电流调制技术，准直0.625 mm，扫描范围：肺尖至膈顶部。对比剂采用优维显（370 mg/mL），经肘静脉放置18 G留置针，A、B双管注射对比剂，注射剂量为85 mL，注射流率3.5 mL/s，之后用相同流率注入15 mL生理盐水冲管。采用Bolus Tracking技术对胸主动脉强化过程进行检测，当强化幅度增至100 HU时，延迟7 s开始触发扫描。采用1.0 mm层厚、0.5 mm层距对扫描数据进行重建后传至工作站进行后处理^[10]^。

### 1.3 图像重建

术前由2名胸外科医师将患者肺部CTA影像DICOM数据导入DeepInsight软件进行肺血管的三维图像重建。分别经过图像加载处理、气管阈值计算、气管提取、血管提取、肺结节提取、调节图像窗宽窗位等步骤，重建3D肺段支气管及动静脉血管模型，并形成3D动画图像，备份并保存至手术室电脑，方便术前主刀医师观察对比。

### 1.4 图像分析

所有患者的CTA三维重建图像均被传入手术室电脑内用于肺段切除术的肺血管分析，由2名主治或以上职称的胸外科医生独立评估切除肺组织的受累肺动脉和肺段间静脉情况。采用5分法判定CTA三维重建图像评估受累肺血管可视化的性能^[11]^（1分：完全不能追踪；2分：部分可追踪；3分：可追踪但亚段血管不清晰；4分：亚段血管可清晰追踪；5分：整个血管可清晰追踪）。同样采用5分法判定CTA三维重建图像辨别、评估受累肺血管效能（1分：完全不能确定；2分：不完全不能确定；3分：模棱两可；4分：可能确定；5分：完全确定）。当医师意见不一致时，参考上级医师的诊断意见协商统一。

### 1.5 手术切除步骤

患者采用可视喉镜行双腔气管导管插管，由同一组手术医师完成手术。手术均顺利完成，无术中并发症。手术切口采用三孔法，第7肋间腋中线1 cm切口置入胸腔镜，主操作孔选择腋前线第4或第5肋间3 cm切口，副操作孔位于第7肋间肩胛下角线与腋后线之间1.5 cm切口。术中解剖肺组织结构中不断对比三维支气管血管成像（three-dimensional bronchoangiography, 3D-CTBA）进行肺结构的辨认，记录所有肺血管解剖，最终由2名胸外科主任医师确认受累肺血管情况。采用丝线结扎细小肺动静脉分支，较大的肺动静脉分支采用腔镜直线切割缝合器切断（强生电动腔镜关节头直线切割吻合器（PSE45, ECR45 M, ECR 45W）。靶段、靶亚段支气管采用腔镜直线切割缝合器切断（强生ECR 45G）。采用“改良膨胀萎陷法” ^[12]^来确定段间交界面，采用锥式肺段切除理念，切割缝合器适形裁剪技术^[12]^分割段间交界面。段间交界面的分离近段门处使用电刀分离（电凝，功率40 W），外周使用腔镜直线切割缝合器进行分割（强生ECR 45 G、ECR 45 G）。使用温无菌蒸馏水在恒定气道压力20 cmH_2_O的情况下测试有无肺漏气。放置单根24 F胸腔引流管连接胸腔闭式引流。所有手术患者术后取标本送病理检测，所有患者的手术切除范围均 > 2 cm。

### 1.6 统计学方法

所有经筛选纳入研究患者的临床数据经汇总，采用*χ*^2^检验比较观察者间评估肺血管的可视化性及辨别血管效能的一致性（k < 0.21为一致性极差，k：0.21-0.40为一致性较差，k：0.41-0.60为中度一致性，k：0.61-0.80为一致性较好，k：0.81-1.00为一致性极好）；使用SPSS 23.0软件进行数据处理及分析，*P* < 0.05被认为其差异具有统计学意义。

## 2 结果

1, 020例预行手术治疗患者均行胸腔镜下精准肺段切除术，手术均顺利完成，无术中转开放手术。患者基本资料见表 1。其中单肺段切除术423例，跨双肺段切除术245例，跨多肺段切除术352例。本次研究中由于操作者不熟练及手术方式难明确等原因，术前共返修3D-CTBA影像42次，经返修更正后，手术规划明确，手术顺利完成。所有患者均在胸腔镜下顺利完成手术。本次研究中，所有患者3D-CTBA成像评估肺血管可视性效能较好（k=0.61-0.80）、术中辨别、评估肺血管效能的一致性极好（k=0.81-1.00）。图 1为一例左上肺固有段切除术通过DeepInsight软件3D-CTBA成像后对比术中发现未显示一根受累血管。

**1:** 患者及肿瘤特征 Patient and tumor characteristics

Variable	*n*
Age (yr)	
< 50	625
≥50	395
Gender (M/F)	496/524
Tumor location:	
R: S1/S2/S3/S4/S5/S6/S7/S8/S9/S10	102/97/84/0/0/88/65/69/46/34
L: S1+2/S3/S4/S5/S6/S8/S9/S10	138/76/5/0/78/53/43/42
Tumor size	
< 1 cm	558
< 2 cm and > 1 cm	462
Pathology	
Benign	98
AAH	225
AIS	386
MIA	264
IAC	47
Operation time (min)	
< 120	585
≥120	435
M: male; F: female; AAH: atypical adenomatous hyperplasia; AIS: adenocarcinoma *in situ*; MIA: microinvasive adenocarcinoma; IAC: invasive adenocarcinoma.	

**1:**
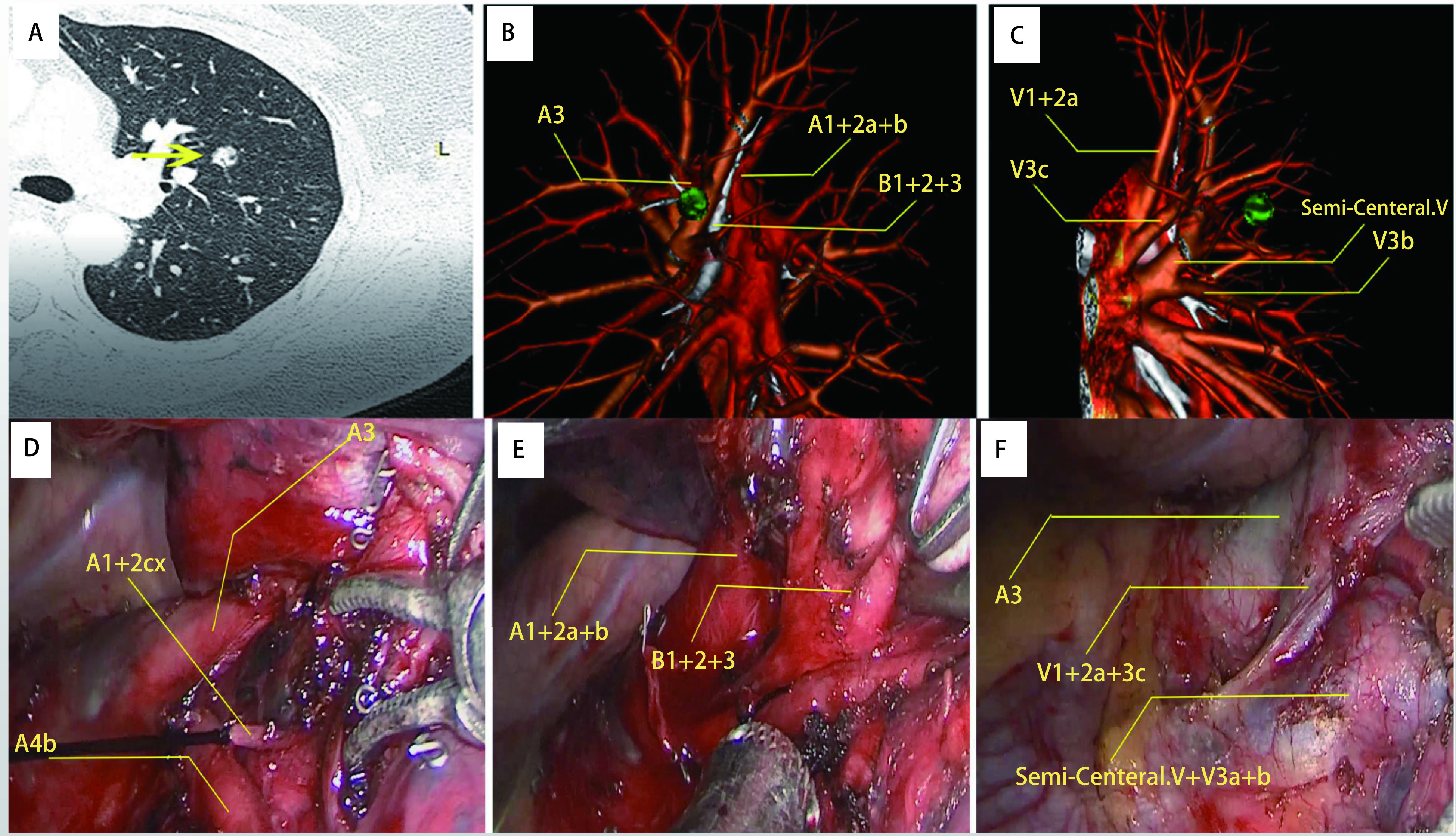
3D-CTBA图像指导下左上肺固有段切除术。A：CT示左上肺叶实性结节（黄色箭头），直径10 mm; B, C：左上肺三维图显示原发病灶（黄色箭头）位于适当肺节段的中心。考虑到结节的性质和位置，切除左侧固有节段可能是最好的选择; D, E, F：术中血管分离图显示目标支气管和血管。术后病理证实为浸润性腺癌（IAC）。图D中白色A1+ 2cx为3D-CTBA图像中未显示的血管。 Illustration of left segmentectomy under the guidance of 3D-CTBA images. A: CT image revealed a solid nodule (yellow arrow), 10 mm in diameter, in the left upper lobe; B, C: The 3D view of the upper left lung revealed the primary lesion (yellow arrow) located in the center of the proper segment. Considering the nature and location of the nodule, resection of the left segment propria may be the best choice; D, E, F: View of the intraoperative separation of the vessels shows the target bronchi and vessels. Postoperative pathologic findings confirmed the diagnosis of IAC. Surgical margin width greater than 20 mm. The white A1+2Cx in Figure D is the blood vessel not shown in the 3D-CTBA image. 3D-CTBA: three-dimensional bronchoangiography; IAC: invasive adenocarcinoma.

### 2.1 DeepInsight软件3D-CTBA评估肺血管可视性效能

若受累的肺组织骑跨2个及以上肺段，则评估这2个及以上的肺段间静脉。本研究共评估肺动脉血管1, 108支，肺静脉血管1, 357支。其观察者间可视性效能一致性较好（表 2）。

**2:** 不同观察者通过3D-CTBA对受累血管的辨别效能评分 Different observers use 3D-CTBA to score the discrimination performance of the involved vessels

Observers	Visual scoring					*K* value
	1	2	3	4	5	
Artery_1_	0	1	2	232	873	0.668
Artery_2_	1	2	1	248	856	
Vein_1_	1	2	3	620	731	0.644
Vein_2_	2	3	1	593	758	
Subscripts 1 and 2 represent the two observation physicians respectively.						

### 2.2 DeepInsight软件3D-CTBA辨别、评估肺血管效能

在本研究中，不同主任医师通过DeepInsight软件3D-CTBA辨别、评估术中受累肺血管效能的一致性极好（表 3）。

**3:** 不同主任医师通过DeepInsight软件3D-CTBA辨别、评估术中受累肺血管效能评分 Different chief physicians use DeepInsight software 3D-CTBA to identify and evaluate the efficacy score of the pulmonary vascular involvement during the operation

Observers	Pulmonary vascular efficacy score					*K* value
	1	2	3	4	5	
Artery_1_	0	1	3	342	762	0.940
Artery_2_	0	2	2	338	766	
Vein_1_	1	2	4	598	752	0.863
Vein_2_	1	1	3	624	728	
Subscripts 1 and 2 represent the two chief observation physicians respectively.						

## 3 讨论

肺段切除术前使用3D-CTBA对受累肺血管情况进行准确评估及术前规划是必要的。在三维影像用于肺部手术之前，肺段间界限难以通过二维CT影像精确区分，位于段间的结节也难以精确其肺段归属。而通过3D-CTBA重建肺血管、支气管和肺结节，可根据肺段/亚段支气管树及肺结节的三维空间位置关系来精准定位肺结节的肺段归属^[13, 14]^，从而达到精准切除受累肺组织，减少肺容积的损失及术后恢复时间^[15]^。此次研究的1, 020例手术患者中，单肺段切除术423例，跨双肺段切除术245例，跨多肺段切除术352例，其中单肺段切除术因手术部位明确、牵涉肺段组织少，其3D-CTBA影像制作简单，段间血管解剖结构清晰，手术方案明确，其观察者间可视性效能一致性与辨别、评估术中受累肺血管效能的一致性极好；跨双肺段切除术的3D-CTBA影像需显示亚段间血管解剖结构、明确双肺段或以上肺组织解剖结构关系，其3D-CTBA影像制作稍复杂，因操作者不熟练其手术方式，共返修3D-CTBA影像12次，其观察者间可视性效能一致性与辨别、评估术中受累肺血管效能的一致性极好；跨多肺段切除术多位于下肺组织，其3D-CTBA影像需显示各种角度下的亚段间及以上的血管结构，由于其肺段间解剖结构关系复杂难定，3D-CTBA影像制作复杂，共返修30次，其观察者间可视性效能一致性与辨别、评估术中受累肺血管效能的一致性较好。综上，DeepInsight软件的3D-CTBA影像对于单肺段切除术及跨双肺段切除术的帮助更好；对于复杂性肺段切除术，术者对于3D-CTBA影像要求较高，要显示亚段及以上的血管结构，需要操作者熟练掌握DeepInsight软件并熟知手术方式及手术路径角度。

DeepInsight为我中心与东软公司共同研发的精准肺部手术辅助软件，因其操作便利、图像清晰、结节定位准确，已于临床大量运用，本研究通过回顾性分析也证明了DeepInsight软件对术中肺部支气管血管的成像是真实及可靠的。DeepInsight软件通过3D-CTBA重建肺血管、支气管和肺结节，可满足绝大部分手术的整体需求，但也存在不足的地方：①由于肺解剖结构的复杂多变，对于患者肺部成像完全后需要裁除健侧肺组织及不相关的肺部结构，以防止其遮挡靶段结构并调节其窗宽窗位以达到最佳观察效果，对不了解患者相关手术的人员来说，可能会造成误裁，从而影响术前手术后规划及术中观察；②由于术中操作是在患者肺组织萎陷状态下完成，而DeepInsight软件是通过清醒状态下患者吸足气后的肺部CTA影像数据完成3D-CTBA重建肺血管、支气管和肺结节的，故即使通过3D-CTBA重建支气管和血管可以清楚地观察到肺部解剖，术前成像仍与术中实际解剖不完全一致，需要操作者术中结合具体的肺血管及支气管走行判断；③DeepInsight软件对于一些极为细小的血管分支（如部分细支气管及终末细支气管间血管结构）或将难以显示清楚，并且不同观察者考虑角度不同从而对其可视性效能与评估术中受累肺血管效能判定不同，故而术中操作者在对靶段肺组织结构进行精密分离时需要具体情况具体分析，必要时需结合二维CT影像对比判断肺组织结构，不能盲目依赖于3D-CTBA成像。

综上所述，DeepInsight软件对术中肺部支气管血管的成像是真实及可靠的，可于术前直观地显示患者肺部结构，帮助主刀医师更好地规划手术路径，规划手术方式。对于经验丰富的临床医生而言，或许仅凭二维CT即可判断病灶位置，明确手术方案，但是对于解剖结构复杂多变的肺部组织结构来说，3D-CTBA成像的引导不但能使之达到准确的节段切除，缩短了手术时间学习曲线，而且对于治疗小恶性肺结节，3D-CTBA成像不仅只有使手术切缘达到安全，还可以尽量减少解剖切除肺组织更有利于保证肿瘤疗效和保留更健康的组织，以达到“圆锥形肺段切除术”的治疗目的。
